# Editorial: Food, nutrition, and diets at net zero. 10 years of Frontiers in Nutrition

**DOI:** 10.3389/fnut.2025.1600417

**Published:** 2025-04-24

**Authors:** Johannes le Coutre

**Affiliations:** ^1^School of Chemical Engineering, University of New South Wales, Sydney, NSW, Australia; ^2^Australian Human Rights Institute, University of New South Wales, Sydney, NSW, Australia

**Keywords:** Frontiers in Nutrition, 10 year anniversary, diets, net zero, nutrition science, sustainability development goals

Ten years ago, in 2014, we launched Frontiers in Nutrition with a bold vision: to create a platform where rigorous science could bridge disciplines, from molecular mechanisms to consumer behavior. We aimed to foster an integrative approach to nutrition science— one that has since gained widespread recognition.

“*No subject pertains more to human life than nutrition*.” This statement from a decade ago still holds true, yet the questions we ask and the challenges we face continue to evolve (1).

Over this past decade, together with an extraordinary team of specialty chief editors, we have not only built a respected journal but also developed 14 specialty sections. These sections—spanning Clinical Nutrition, Food Chemistry, Nutrition and Microbes, Nutritional Epidemiology, and more—have become hubs of leadership in their respective fields. Every 5 years, our flagship editorial series, *Goals in Nutrition Science* (2, 3), has reflected the shifting landscape of the discipline, tackling topics from functional foods and nutrition methodologies to food security, ultra-processed foods (4), and cellular agriculture (5, 6).

As we enter our second decade, new frontiers await. The impact of artificial intelligence, the role of GLP-1 receptor agonists such as semaglutide (7), the growing concern over plastics in food (8), and the ever-present challenges of food security demand our attention. With the UN's Sustainable Development Goals set to conclude in 2030, we mark this milestone by presenting fresh perspectives on nutrition's role in shaping a future of zero hunger and net-zero emissions. This anniversary is not just a celebration—it is a call to action.

The review article “*The role of algae, fungi, and insect-derived proteins and bioactive peptides in preventive and clinical nutrition*” explores the potential of algae, fungi, and insect-derived proteins and their bioactive peptides in nutrition (Yimam et al.). These alternative proteins offer sustainable, nutrient-rich sources with health benefits such as antimicrobial, anti-inflammatory, and antioxidant properties. Algae-derived peptides show promise in managing hypertension, obesity, type 2 diabetes, and certain cancers. Fungi, comprising mushrooms and derived mycoproteins, provide essential nutrients and may aid in cholesterol reduction, immune support, and metabolic health. Insects, rich in protein and essential amino acids, have been studied for antihypertensive, antimicrobial, and antioxidant effects. The review emphasizes the importance of bioactive peptides in modulating physiological functions and their potential in clinical nutrition. However, more human trials are needed to confirm benefits and determine dosages. The authors highlight research gaps in bioavailability, consumer acceptance, and regulations. Addressing these gaps could advance functional foods and sustainable diets, supporting both preventive and clinical nutrition.

The perspective article “*Diet, nutrition, and climate: historical and contemporary connections*” by Demmler and Tutwiler examines the links between nutrition and climate change, emphasizing integrated strategies for health and sustainability. Despite progress, micronutrient deficiencies persist, especially in Sub-Saharan Africa and South Asia. Food systems contribute to environmental degradation through deforestation, greenhouse gas emissions, and excessive water use. The authors highlight the need to integrate nutrition into climate policies and to strengthen food system resilience. Initiatives like the “Scaling Up Nutrition” movement and the “Food Systems Dashboard” help transform food systems, but stronger policies are required to align nutrition, climate, and equity goals. The paper calls for increased investment, better policy frameworks, and innovations in technology and data monitoring. Promoting biodiversity, addressing micronutrient deficiencies, and developing climate-smart crops are essential for sustainable agriculture. The authors reiterate that achieving resilient and equitable food systems requires collaboration across sectors, ensuring long-term solutions for both nutrition and climate challenges.

The perspective article “*Integrating food is medicine and regenerative agriculture for planetary health*” explores the intersection of Food is Medicine (FIM) and regenerative agriculture (RA) to improve health and sustainability (Rahman et al.). FIM initiatives, like produce prescriptions and medically tailored meals, promote nutrition equity, while RA enhances soil health, biodiversity, and reduces synthetic inputs. Current food systems contribute to greenhouse gas emissions, water use, and biodiversity loss while driving diet-related diseases. Integrating FIM and RA offers a synergistic approach to improving individual health and ecological wellbeing. The paper highlights real-world examples of RA practices in FIM programs, addressing challenges such as crop selection and logistics in rural areas. The authors call for collaboration among policymakers, healthcare providers, farmers, and researchers to strengthen links between agriculture and healthcare. Clear definitions of RA and stronger partnerships could create resilient food systems and improve health at individual and population levels, advancing planetary health.

In their original research study entitled “*The protein transition: what determines the animal-to-plant (A:P) protein ratios in global diets*” Drewnowski and Hooker examine global shifts in animal-to-plant (A:P) protein ratios, influenced by income levels. In high-income countries (HICs), about 65% of dietary protein comes from animal sources, with efforts underway to lower this for health and sustainability. In contrast, low- and middle-income countries (LMICs) are increasing animal protein consumption as incomes rise, following “Bennett's Law”. Data from the FAO and World Bank show a strong correlation between national income and A:P ratios. By 2020, HICs had A:P ratios above 60:40, while countries like Brazil and China saw significant increases. The study highlights the challenge of global dietary recommendations, as HICs shift toward plant-based diets while LMICs increase animal protein intake. The authors stress that policies to reduce A:P ratios in HICs must account for economic forces shaping diets worldwide, ensuring strategies balance health, sustainability, and economic development across different regions.

Another research article, “ *Ecological impact and the metabolic food waste of overweight and obese adult individuals living in North European and Mediterranean countries*”, investigates the environmental impact of overeating through the concept of Metabolic Food Waste (MFW), which quantifies excess food consumption leading to excess body fat (EBF) and its ecological costs (Angelino et al.). Overeating not only harms health but also increases greenhouse gas emissions, water use, and land requirements for food production. The study analyzes dietary patterns in North European and Mediterranean countries, revealing how surplus food intake contributes to environmental degradation. Addressing overeating could reduce GHG emissions, conserve water, and promote sustainable land use. The research underscores the need for integrated strategies that link healthier eating habits with environmental sustainability, highlighting the interconnectedness of nutrition and ecological wellbeing.

Finally, the review article “*Africa's contribution to global sustainable and healthy diets: a scoping review*” explores Africa's traditional diets and their role in health and environmental sustainability (Oniang'o et al.). Rich in whole grains, legumes, and indigenous foods, these diets support nutrition and agroecological practices that enhance biodiversity and soil health. However, dietary transitions toward processed, Westernized foods threaten food system independence and increase non-communicable diseases. Dependence on imported foods also heightens vulnerability to global market fluctuations, affecting food security. The review highlights the value of preserving traditional African diets to promote nutrition, sustainability, and food sovereignty. It calls for policies that integrate indigenous food systems into global health and environmental strategies, emphasizing Africa's contribution to sustainable diets.
